# SF_6_ High-Voltage Circuit Breaker Contact Status Detection at Different Currents

**DOI:** 10.3390/s22218490

**Published:** 2022-11-04

**Authors:** Ze Guo, Linjing Li, Weimeng Han, Zixuan Guo

**Affiliations:** 1 State Key Laboratory of Reliability and Intelligence of Electrical Equipment, Hebei University of Technology, Tianjin 300401, China; 2 Key Laboratory of Electromagnetic Field and Electrical Apparatus Reliability of Hebei Province, Hebei University of Technology, Tianjin 300401, China

**Keywords:** SF_6_ HVCB, non-destructive testing, contact status detection, vibration signal, DNN, Bayesian optimization

## Abstract

Currently, the online non-destructive testing (NDT) methods to measure the contact states of high-voltage circuit breakers (HVCBs) with SF_6_ gas as a quenching medium are lacking. This paper aims to put forward a novel method to detect the contact state of an HVCB based on the vibrational signal. First, for a 40.5-kV SF_6_ HVCB prototype, a mechanical vibration detection system along with a high-current generator to provide the test current is designed. Given this, vibration test experiments are carried out, and the vibration signal data under various currents and corresponding contact states are obtained. Afterward, a feature extraction method based on the frequency is designed. The state of the HVCB contacts is then determined using optimized deep neural networks (DNNs) along with the method of adaptive moment estimation (Adam) on the obtained experimental data. Finally, the hyperparameters for the DNNs are tuned using the Bayesian optimization (BO) technique, and a global HVCB contact state recognition model at various currents is proposed. The obtained results clearly depict that the proposed recognition model can accurately identify five various contact states of HVCBs for the currents between 1000 A and 3500 A, and the recognition accuracy rate is above 96%. The designed experimental and theoretical analysis in our study will provide the references for future monitoring and diagnosis of faults in HVCBs.

## 1. Introduction

Mechanical vibrations that occur in HVCBs during the current interruption operation can be collected by installing acceleration sensors on the HVCB housing. The obtained vibration signals are then used to identify the mechanical state of the HVCB [1,2,3]. In this process, due to the influence of the HVCB’s flow size, field noise and the complex structure of the HVCB itself, the acquired vibration signal may be distorted, which increases the difficulty of finding an accurate and efficient contact state recognition model [4]. An HVCB’s contacts will wear out under the impact of the mechanical closing force and contact stress during current interruption, resulting in contact deterioration [5,6]. Establishing a state-of-the-art HVCB contact status identification model can reduce its maintenance costs and avoid potentially devastating consequences before a severe accident occurs [7,8].

At present, the contact status of an HVCB can only be identified by measuring its contact resistance before the equipment is put into operation, and there are only a few research studies that are available on the online contact state identification of in-service HVCBs. The increasing studies on vibration detection methods in NDT for identifying the conditions of industrial equipment (e.g., applications in bearings) are attracting significant attention, owing to their optimized and controlled strategies. For example, scholars have studied the fault diagnosis methods of bearings based on vibration signals, including wavelet packet analysis [9], Complete Ensemble Empirical Mode Decomposition with Adaptive Noise (CEEMDAN) [10], the Teager–Kaiser Energy Operator [11], support vector machines (SVMs) [12,13], neural networks (NNs) [14,15] and enhanced differential product weighted morphological filtering [16]. Given this, it was observed that when the HVCB fails, there is also an abnormal vibration signal which can be used to analyze the HVCB’s running state. In this regard, scholars have put their efforts toward analyzing the HVCB vibration signal for HVCB fault diagnosis [17,18,19,20,21]. Previous studies have indicated that the contact mode of an HVCB changes due to deteriorated contacts [22,23,24]. The flowing current through these poor contacts will cause the abnormal vibration of HVCBs. In actual practice, it is necessary to achieve real-time and efficient monitoring and judgment of the contact state of an HVCB at different currents, and it is necessary to further explore the relationship between the vibration signal and the contact state of an HVCB. In terms of HVCB fault recognition, this mainly includes three types of methods that are based on qualitative empirical knowledge, statistical analysis and artificial intelligence (AI) techniques. The deployment of the first two methods is difficult to implement, owing to the complex structure of HVCBs, the strong uncertainty and the large amount of required data. Currently, experts and scholars generally use AI algorithms to achieve fault diagnosis of HVCBs to overcome the above problems, and the algorithms used in fault diagnosis of HVCBs mainly include support vector machines (SVMs) [25,26,27], neural networks (NNs) [28,29], clustering [30], random forest [31], autoencoder networks [32] and so on. The previously presented fault recognition model for HVCBs mostly uses manual parameter adjustment methods for hyperparameter optimization. The learning rate of NNs, the number of hidden layers and the number of neurons in each layer may be problem-specific and related to the complexity of the data. Manual parameter adjustment methods are used to set a fixed learning rate, the number of hidden layers, the number of hidden layer nodes and the dropout rate for the model, which are difficult to adapt due to the complex and varying working conditions of the actual HVCB [33,34,35]. Therefore, based on this problem, it is an essential need to establish an NN recognition model with hyperparameter optimization for detecting the contact state of the HVCB while considering various currents.

Due to its remarkable arc-quenching capability and dielectric insulating properties, SF_6_ is widely used as a switching and insulating gas in high-voltage apparatuses [36,37]. In addition, it is chemically inert, non-toxic and non-flammable [38]. Therefore, using SF_6_ as the quenching medium, an HVCB with compact structure, strong environmental adaptability and high working reliability can be manufactured. Nevertheless, SF_6_ has the major drawback of presenting a global warming potential (GWP) of 23,500 [39]. The search for an alternative gas for SF_6_ continues unceasingly. Research was conducted in the past few decades on substitutes to SF_6_, covering different candidates including common gases (nitrogen, air and CO_2_) and vacuum and gas mixtures [40]. Traditional gases such as dry air, nitrogen, CO_2_ or their mixtures have the advantage of a low global warming potential but very limited dielectric strength compared with SF_6_. A vacuum is widely used in the medium voltage domain as a current interruption medium, but the application of a vacuum at high voltages does not appear to be economically competitive. Moreover, mixtures of SF_6_ and other gases such as SF_6_-CF_4_, SF_6_-C_2_F_6_ and SF_6_-N_2_ are also considered potential SF_6_ substitutes [41,42,43,44,45]. They can be used to reduce costs and the environmental impact of SF_6_ gas to a certain extent. At present, HVCBs with SF_6_ gas as a quenching medium will still occupy a large share of the market for a long time. In this paper, we chose SF_6_ HCVB as our research object.

In this paper, a feature extraction method based on frequency is proposed, a recognition model composed of a DNN and BO is constructed, and an NDT for the contact state of the SF_6_ HVCB is proposed.

The main contributions of this paper can be summarized as follows:

1. The vibration test platform of the SF_6_ HVCB is set up, and the vibration signal of the HVCB in different contact states is obtained while considering a current between 1000 A and 3000 A.
2. Using the method of a DNN, the amplitudes of the signal in frequency multiples of 50 Hz are used as the eigenvalue matrix to complete the recognition of the contact state of the HVCB.
3. The BO is used for hyperparameter optimization of the NNs so that the proposed model can be applied globally to SF_6_ HVCB while considering different currents while having a wider range of applications.

The remainder of the paper is organized as follows. Section 2 explains the experimental platform of the HVCB vibration test, experimental procedures, vibration mechanism and vibration signal analysis. The HVCB contact status recognition model is presented in Section 3. The model recognition results and discussions are presented in Section 4. Section 5 concludes the paper.

## 2. Experiments and Vibration Data Analysis

### 2.1. HVCB Vibration Detection Experimental System

**Figure 1:**
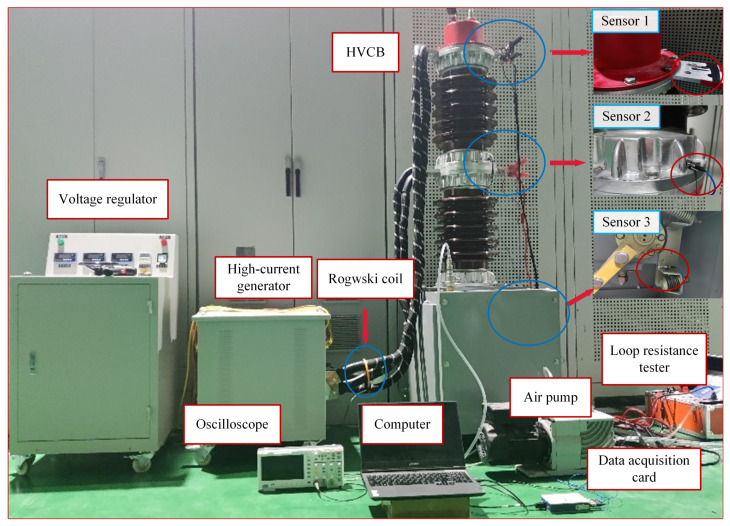
Experimental platform structure diagram.

Vibration detection in an HVCB was carried out by considering a prototype design of a 40.5-kV SF_6_ HVCB as shown in the experimental platform in Figure 1. First, a large current was injected from both ends of the HVCB interface using a high-current generator consisting of a voltage regulator with a maximum output current of 5000 A and a single-phase output. The maximum applied current in this study was 3500 A. The frequency of the applied current in the experiment was 50 Hz. The current through the HVCB was then measured using a Rogowski coil connected with an oscilloscope. In the experiment, a Rogowski coil was sheathed on an energized conductor to measure the amount of current flowing into the HVCB. The lead wires at both ends of the HVCB were connected in parallel with multiple strands of copper conductors, and the total through-current cross-sectional area was greater than 1000 mm^2^. The contact state of the HVCB was changed by manually adjusting its tie rod, and the specific value of the contact resistance was confirmed by the loop resistance tester. SF_6_ gas was injected into the HVCB using an air pump with a pressure of 0.35 MPa.

A high-quality and high-precision data acquisition system was required for the subsequent signal analysis and feature extraction. The vibration signal acquisition platform consisted of signal detection sensors, a data acquisition card and a computer to process the data. The various parameters of the acquisition platform are summarized in Table 1. Considering the frequency and amplitude of the mechanical vibration, as well as the strong magnetic field for in-service HVCBs, acceleration sensors with reliable performance from a PCB company were selected. For the more precise and comprehensive detection of the vibration signal in the HVCB, the acceleration sensors were connected at three different positions and numbered as 1, 2 and 3. The different positions of the sensors were as follows: (1) in front of the HVCB contact, (2) in front of the tie rod and (3) near the operating assembly. These sensors at different positions expanded the fault monitoring range and could effectively collect the vibration signals near the contact of the HVCB, the operation link, the insulator and other different parts. The obtained data from the sensors were transferred to the data acquisition card (NI9234) and recorded on a computer for further processing.

### 2.2. HVCB Vibration Detection Experimental Procedures

First, the HVCB was filled with SF_6_ gas at a pressure of 0.35 MPa. Secondly, the contact state of the HVCB was changed by manually adjusting the HVCB tie rod, and the specific value of the contact resistance was confirmed with a loop resistance tester. At this time, the HVCB contact was adjusted to a good contact state, and the contact resistance of the HVCB was measured with a loop resistance tester to be 48 μΩ. Thirdly, the HVCB vibration signal at 6 various currents between 1000 A and 3500 A with an incremental step of 500 A when the HVCB was in good contact was measured using sensors placed on the HVCB in advance. In order to ensure the safety of the experiment and ensure that the data were not affected, after each group of experiments was completed, we stopped the current flow, paused for 15 min and then started the next group of experiments. The above is the acquisition of vibration signals when the HVCB contacts were in good contact under six currents.

Next, we adjusted the HVCB tie rod appropriately to make its contact state a slightly poor contact state. The contact resistance was measured to be 78 μΩ. At this time, the vibration signal of the HVCB at 6 various currents between 1000 A and 3500 A with an incremental step of 500 A was measured and recorded. By analogy, the subsequent experiments for moderate contact defects (contact resistance of 93 μΩ), severe contact defects (contact resistance of 117 μΩ) and extremely severe contact defects (contact resistance of 150 μΩ) were continued. A total of 5 experiments under different contact states were completed, and 6 experiments at different currents were completed in each contact state, resulting in 30 sets of experimental data. The detection time of each set of data was 45 s, and the sampling frequency was set to 51.2 kHz.

### 2.3. HVCB Vibration Characteristics

The excitation forces of the housing during the operation of the HVCB are the mechanical forces, the electromagnetic forces and the discharge forces. This study discusses the vibration signal of the in-service HVCB and does not consider the phenomenon of the HVCB closing and discharging. Therefore, only the vibration signal generated by the electromagnetic force was considered. The electrical power between the contacts and the electrical forces received by the metal housing of the HVCB were calculated separately, and their frequencies were analyzed.

First, we calculated the electrical power received at both ends of the contact. When there is a current flowing through the equipment circuit, the current line shrinks near the contact surface of the HVCB to generate electrical power, which causes the equipment to vibrate. Suppose that the current passing through the contacts is $i=I_{0}\cos(\omega t)$, where ω is the angular frequency corresponding to the power frequency. The electrical force between the contacts can be calculated using the following equation:

(1) $$F_{1}=\frac{\mu_{0}}{4\pi }i^{2}\ln\frac{D}{2a}=\frac{\mu_{0}I_{{}_{0}}^{2}\left\{\cos(2\omega t)+1\right\}}{8\pi }\ln\frac{D}{2a}$$

The parameter *a* in the above equation can be calculated using the following equation:

(2) $$a=\sqrt{\frac{F_{j}}{\pi \zeta H_{b}}}$$

where *D* is the diameter of the contact surface, *a* is the radius of the contact spot, *F_j_* is the initial pressure acting on the contact, ζ is the material deformation coefficient between 0.3 and 1 and *H_b_* is the Brinell strength of the transparent material.

Therefore, under normal circumstances, the force on the HVCB contact is a simple harmonic force with twice the frequency of the power supply so that the vibration of the HVCB contact caused by the electromagnetic force is a vibration of twice the frequency of the power supply.

Subsequently, the electrical power of the metal housing of the circuit breaker is calculated. Suppose that *R* is the diameter of the cylindrical shell of the HVCB, *R_eq_* is the equivalent resistance of the current loop, $\mu_{0}$ is the vacuum permeability and $\mu_{r}$ is the relative permeability. *B_R_* is the magnetic induction strength of the metal cell when the contact is introduced with the current. Assuming that the base area of the metal unit divided into HVCB shells is *S* (where *S* approaches zero), the length is *L*, the metal element is treated as a thin shell, and the electromagnetic force on the edge of the metal element divided by the HVCB shell is approximately expressed as follows:

(3) $$F_{2}=B_{R}iL=\frac{-\mu_{0}^{2}\mu_{r}^{2}I_{0}^{2}S\omega L}{8\pi R^{2}R_{eq}}\sin(2\omega t)$$

Therefore, under normal circumstances, the combined force of each metal unit is a simple harmonic force with twice the frequency of the power supply so that the vibration of the HVCB shell caused by the electromagnetic force is a vibration of twice the frequency of the power frequency.

From the above theoretical calculations, when the current into the HVCB is 50 Hz, and the HVCB is in normal operation, the frequency of the vibration signal collected from the HVCB should be 100 Hz.

### 2.4. Vibration Data Analysis

**Figure 2:**
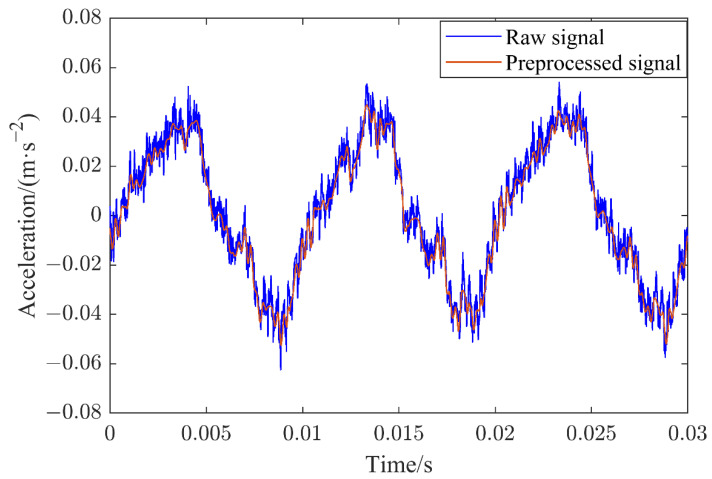
Vibration signal comparison before and after preprocessing.

The contact state recognition process for the SF_6_ HVCB in this study included three steps: vibration signal preprocessing, feature extraction and the establishment of a contact state recognition model. Due to the influence of onsite high electromagnetic interference and the complex structure of the HVCB itself, the collected vibration signal may have contained a certain amount of noise that needed to be filtered for precise and comprehensive measurements. Figure 2 depicts the comparison of the vibration signal acquired by sensor 1 at 2000 A before and after preprocessing when the contact state of the HVCB contact was in good condition. It can be observed that the vibration signal was a stationary signal whose frequency did not change with time, which is the main reason for the subsequent selection of Fourier analysis. However, the vibration signal was not stable around the zero point of vibration, because during the operation of the HVCB, there was heat generated, making the sensors appear to have the “zero drift” phenomenon. Therefore, the DC component in the signal needed to be removed in subsequent signal processing to eliminate the impact of “zero drift” on the data analysis.

**Figure 3:**
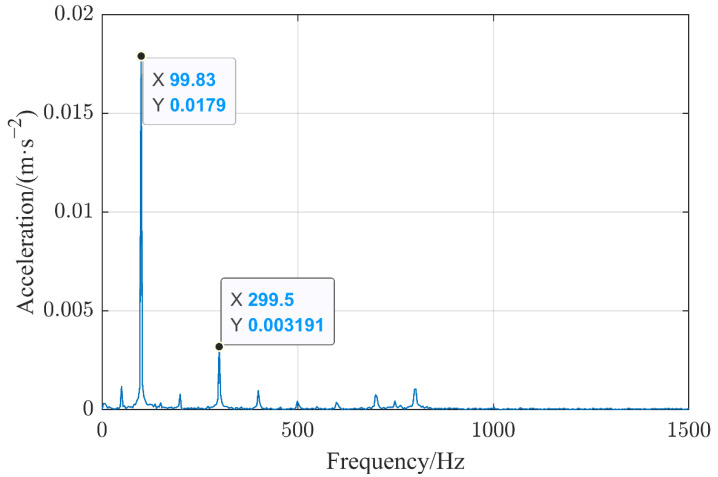
Spectrum of the vibration signal.

Figure 3 is a spectrum of the vibration signal collected by sensor 1 at 2000 A when the contact state of the HVCB contact was in good condition and was decomposed by Fourier analysis. The vibration signal frequency of the collection point was concentrated at integer multiples of 50 Hz, the range was 50–1500 Hz, and the amplitude was the largest at 100 Hz, which verifies that the vibration was mainly caused by electromagnetic force, and the frequency of the electromagnetic force was 100 Hz. At the same time, it can be seen that the frequency of the vibration signal was mainly 99.83 Hz instead of the accurate 100 Hz, and it was caused by the on-site high electromagnetic interference as well as the frequency of the power supply voltage. For subsequent feature extraction, the amplitudes of the signals with frequencies near integer multiples of 50 Hz were selected as the eigenvalue. In order to avoid missing some feature points due to frequency shifting, in the actual calculation, the point with the largest amplitude was selected in the range of (50×k ± 5) Hz (where k is a positive integer between 1 and 30), and their horizontal and vertical coordinate values were recorded. This was used as the eigenvalue to form a matrix of eigenvalues.

**Figure 4:**
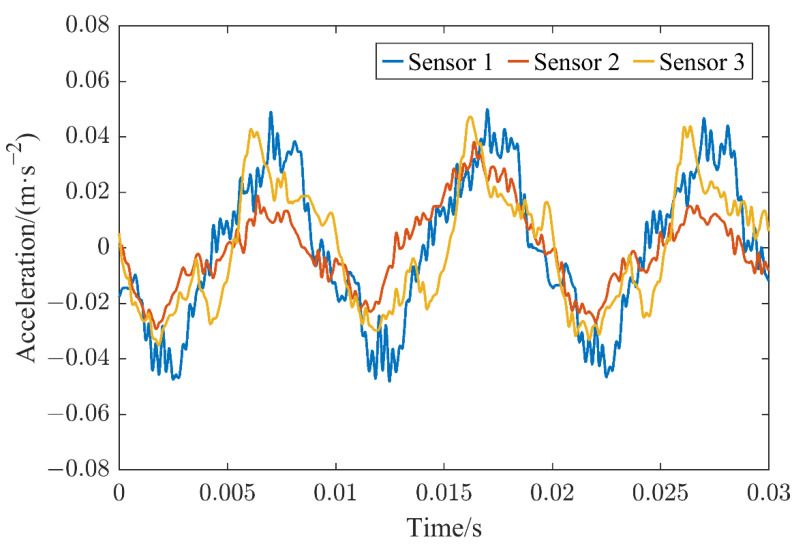
Time domain diagram of the vibration signals acquired by different sensors when the contacts were in good contact.

**Figure 5:**
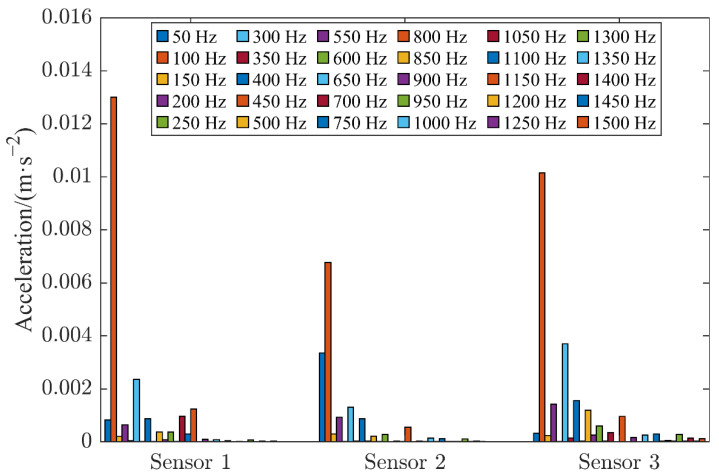
Frequency domain diagram of vibration signals acquired by different sensors when contacts were in good contact.

The preprocessed time domain and frequency domain patterns of the vibration signals obtained by the three sensors are illustrated in Figure 4 and Figure 5, respectively. The vibration signals were obtained at a current of 2000 A when the HVCB contacts were in good condition with a contact resistance of 48 μΩ. In Figure 5, the 3 sets of histograms represent sensors 1–3, and the columns in a set of histograms represent the amplitude of the signal collected by the sensor when the signal’s frequency was 50–1500 Hz with an integer multiple of 50 Hz. It can be observed that the vibration amplitudes obtained by sensors 1 and 3 were much higher compared with the vibration amplitude obtained by sensor 2. In the actual measurement, it was found that the vibration signal obtained by sensor 3 could easily be affected by the operating mechanism. In addition, it contained a multi-frequency signal and richer information on noise that may bring severe challenges for the analysis and processing of the signal. Based on comprehensive consideration, sensor 1, which was closer to the contact, making it easier to analyze its resulting vibration signal, was selected as the subsequent analysis object.

**Figure 6:**
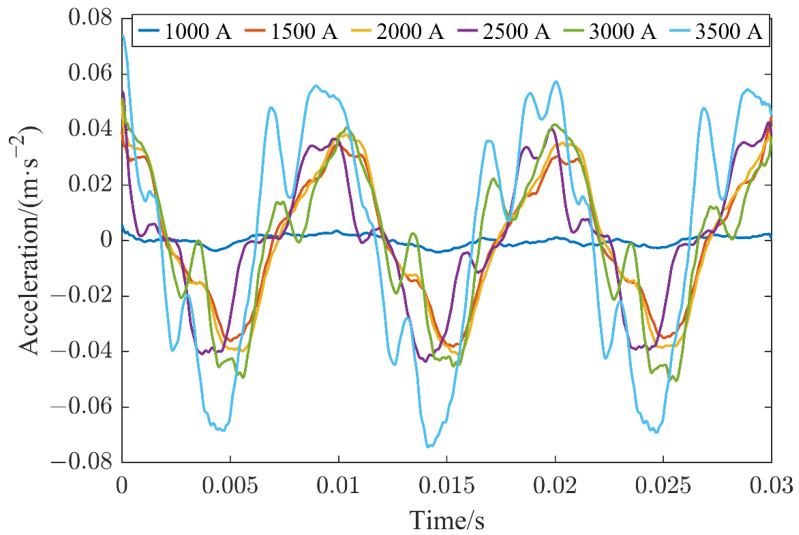
Time domain diagram of the vibration signals in good contact states acquired at different currents.

Figure 6 shows a comparison of the time domain of the vibration signal collected when the contact of the HVCB was in good condition at a current between 1000 A and 3500 A with an incremental step of 500 A. The data used herein were measured by sensor 1, as already mentioned above. After that, the obtained data were filtered and smoothed in order to analyze the results more clearly. It can be observed from Figure 6 that the vibration signal of the HVCB increased nonlinearly with the increase in the current.

**Figure 7:**
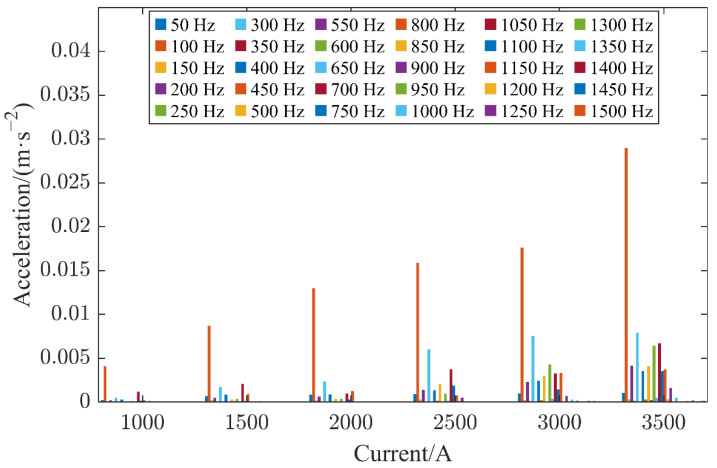
Frequency domain diagram of vibration signals in good contact state acquired at different currents.

Similarly, Figure 7 shows a comparison of the frequency domains of the vibration signals collected when the contact of the HVCB was in good condition at a current between 1000 A and 3500 A with an incremental step of 500 A. The amplitude of the vibration signal was augmented dramatically with the increase in the current. However, the ratio of the signal amplitudes at each frequency was not static, and the comparison was most obvious from 2000 A to 3000 A. In this current range, the amplitude of the signal at a frequency of 100 Hz increased slightly, while the amplitude of the signal at 300 Hz rose rapidly. Given this, it is not feasible to say directly that for different current situations, the amplitude of the signal at each frequency is enough to determine the contact of the current HVCB. Therefore, it is necessary to introduce deep learning methods to find potential internal laws.

**Figure 8:**
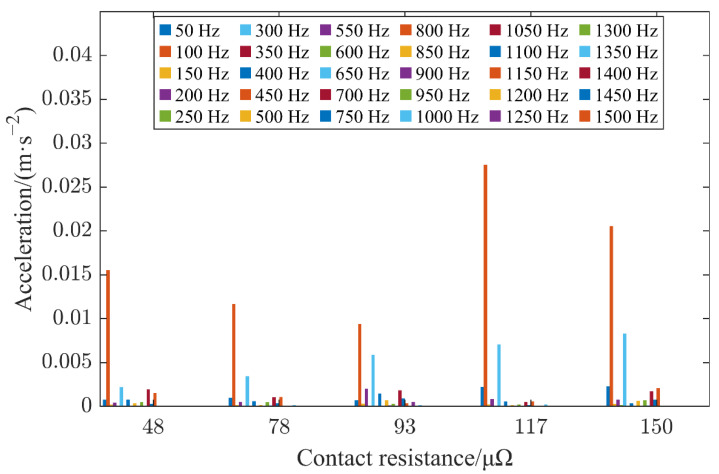
Frequency domain plot of vibration signals in poor contact conditions.

As shown in Figure 8, the contact resistance was changed by adjusting the angle of the HVCB tie rod that was confirmed by the circuit resistance tester. Figure 8 shows the vibration signal collected by sensor 1 at 2000 A when the contacts of the HVCB were in poor condition with contact resistances of 78 μΩ, 93 μΩ, 117 μΩ and 150 μΩ. It is worth mentioning that in the different contact states of the HVCB, the frequency obtained by Fourier decomposition was basically located between 0 and 1500 Hz, and the amplitude at 100 Hz held the highest value. Additionally, there were some variations in the signal amplitude near 50–1500 Hz with integer multiples of 50 Hz. As the contact state deteriorated, the 100-Hz signal amplitude showed a fluctuating change, while the 300-Hz signal amplitude increased slightly. In short, the amplitude changes of different frequencies showed a certain law, but it was difficult to explore its specific law based on a simple calculation. Moreover, under actual working conditions, the current magnitude and contact resistance of the HVCB have complex and variable relations, and the amount of data is also larger, making them difficult to process using simple algorithms. Therefore, it is an essential need to further excavate the potential deep information of the vibration signal in order to conduct a more accurate and comprehensive analysis.

## 3. HVCB Contact Status Recognition Model Based on DNNs

### 3.1. Deep Neural Networks (DNNs)

**Figure 9:**
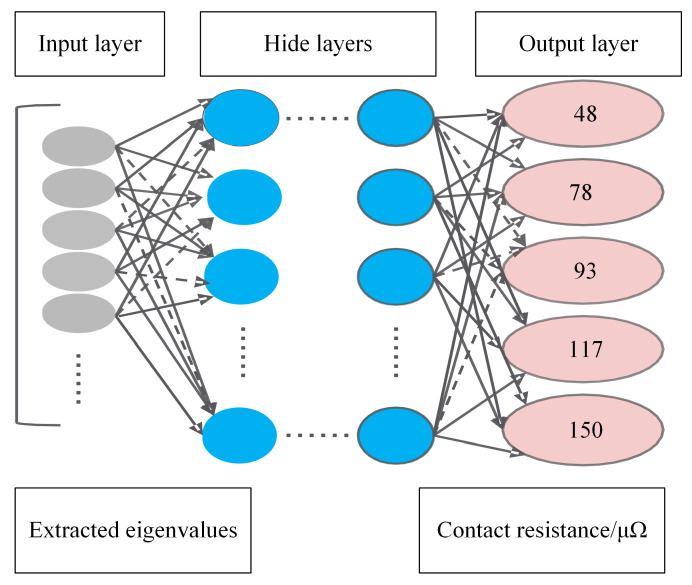
DNN architecture diagram.

A DNN is a feedforward artificial NN that differs from general NNs by having multiple layers of hidden units between the inputs and outputs. DNNs utilize many properties of the natural signal that are combinatorial hierarchies, where higher-level features are obtained by combining lower-level features [46]. DNN are divided according to the position of different layers, and can be divided into the following three categories; (i) the input layer, (ii) the hidden layer, and (iii) the output layer as shown in DNN structure diagram in Figure 9. In this study, the input parameters for the input layer are the amplitude of the extracted frequency signal, the number of hidden layers, (Hidden layer nodes will be adjusted according to the model recognition.) and the output layer, representing five classification results. The dotted line represents an idea of preventing the NN from overfitting by randomly removing elements (along with their connections) from the NN during training, which can be quantified by the dropout rate. Similarly, the dropout rate is subsequently adjusted according to the identification situation.

Forward propagation calculates the predicted value and then calculates the loss based on the difference between the predicted value and the true value, and backpropagation updates the parameters of each layer sequentially from the last layer forward according to the loss function. In the forward propagation phase, the hidden layer takes the output of the previous layer as the input of the latter layer:

(4) $$b_{k}^{L}=\sum_{i=0}^{n}w_{ik}^{L}\times a_{i}^{L-1}+d_{k}^{L}$$

(5) $$a_{k}^{L}=f(b_{k}^{L})$$

In this equation, $b_{k}^{L}$ and $a_{k}^{L}$ represent the outputs of the *k* neuron in the *L*th layer of the DNN before and after activation, respectively, $w_{ik}^{L}$ represents the linear transfer coefficient from $a_{i}^{L-1}$ to $b_{k}^{L}$, $d_{k}^{L}$ represents the bias constant of the $b_{k}^{L}$ forward propagation function and *f*(*x*) is the activation function. As a classification model, the Sigmoid function was chosen as the activation function. The mathematical expression for the Sigmoid function is as follows:

(6) $$f(x)=\frac{1}{1+e^{-x}}$$

Binary cross-entropy is often used as a loss function for classification problems, where cross-entropy is used as a loss function to assess the losses of the classification models in the prediction process. When using small batch gradient descents, this updated selection of weights leads to the risk of the loss function stagnating at the local minimum. The adaptive moment estimation (Adam) algorithm can be used to solve the optimization problem of large data volumes and high feature latitudes, and it requires only a small amount of memory in machine learning [47].

### 3.2. Bayesian Hyperparameter Optimization (BO)

Common automated machine learning hyperparameter methods are mesh tuning, random search and BO. The former is slower. The latter, while faster, is more likely to miss important points in the search space during processing. The BO algorithm establishes a substitution function based on the past evaluation results of the objective function to find the value of the minimized objective function. Therefore, BO is comparatively much faster, smaller in terms of iterations and more efficient [48,49,50]. In this paper, the BO based on a tree-structured Parzen estimator (TPE) with a good effect and speed in a high-dimensional space was used as a method of automatic machine learning hyperparameter optimization for DNNs.

BO involves an iterative process along with the proxy function and the acquisition function as its two main components. In each iteration, a probabilistic proxy model is built using TPE:

(7) $$p(x|y)=\left\{ \begin{array}{l} l(x),y<y^{*}\\ g(x),y\geq y^{*} \end{array}\right.$$

In the equation, *x* is the observation point obtained in the search spaces, y* = min{(*x*_1_, *f*(*x*_1_)), …, (*x_i_*, *f*(*x_i_*))} represents the optimal value on the observation domain, where *l*(*x*) is the density formed using the observation {*x_i_*} that results in the corresponding loss *f*(*x_i_*) being less than *y*^∗^, and *g*(*x*) is the density formed using the observed value {*x_i_*} to make the corresponding loss *f*(*x_i_*) greater than *y*^∗^.

In the TPE, the expected improvement (EI) is used as the acquisition function, and the next evaluation point for the objective function value is optimized until the maximum number of iterations is reached.

In the equation, *p*(*x*) is the probability of reaching an observation point *x*, and *p*(*y*) represents the probability that *y* is the optimal value. The equation for the calculation of the EI is as follows:

(8) $$EI_{y^{*}}(x)=\int_{-\infty }^{y^{*}}\left(y^{*}-y)p(y|x\right)dy=\int_{-\infty }^{y^{*}}\left(y^{*}-y\right)\frac{p(x|y)p(y)}{p(x)}dy$$

### 3.3. Recognition Process

**Figure 10:**
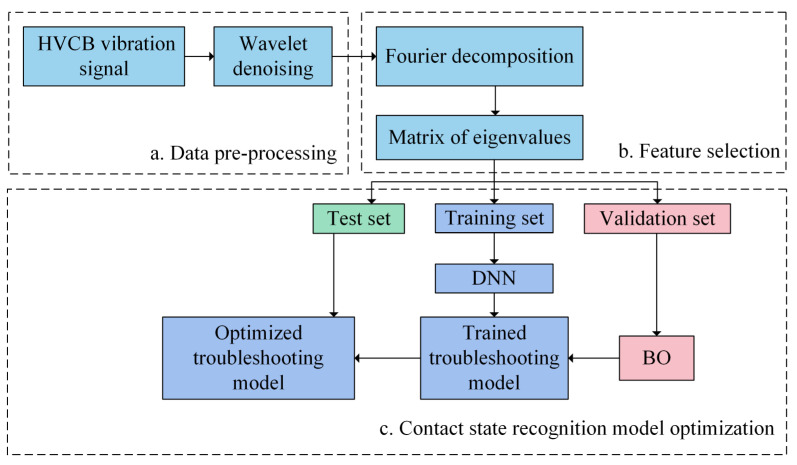
Contact state recognition model flowchart.

The hyperparameter combination after the iteration was used as the optimal input parameter combination to enter the HVCB contact state recognition model and to complete the HVCB contact state recognition. The flowchart of the HVCB contact state recognition model is shown in Figure 10.

The 45-s vibration signals obtained by each set of experiments, or 2,304,000 sample points, were evenly divided into 768 sets of data. They were analyzed separately by Fourier decomposition, and the amplitude of the signal with a frequency of 50 times between 0 and 1500 Hz was saved, forming a matrix of 768 × 30, which would be fed into the DNN as eigenvalues for the next step of classification.

Based on this, the number of input layer nodes for the DNN obtained was 30, and the number of output layer nodes was 5. The experimental data were divided into three parts: a training set, a validation set and a test set, where 70% of the data in the samples in each state was randomly selected as the training set, while 20% was the verification set and 10% was the test set. The training set was used to train a DNN model. The validation set was used for BO to find suitable hyperparameters. Finally, the test set was input into the final DNN model to test the accuracy of the recognition model and verify the effectiveness of the recognition model.

## 4. Recognition Results and Discussions

Table 1 shows the accuracy of the state identification at different currents when using fixed parameters without BO. As can be seen in Table 2, when the DNN adopted fixed parameters, it was impossible to guarantee a high recognition rate for each current condition. At the same time, manual parameter adjustment took a long time and was inefficient, which could limit its usage for practical applications. Therefore, it is necessary to find more efficient hyperparameter optimization methods.

Using BO, one can set the search space and search time in advance, which can greatly improve the accuracy of the recognition model. In the actual experiment, the manual parameter adjustment method was first used to determine the optimization space according to the recognition accuracy under a combination of different hyperparameters. The optimization space set in this study is illustrated in Table 3.

As shown in Table 4, at the current between 1000 A and 3500 A, the recognition model could obtain a satisfactory accuracy rate and accurately identify the five contact states of the HVCB.

**Figure 11:**
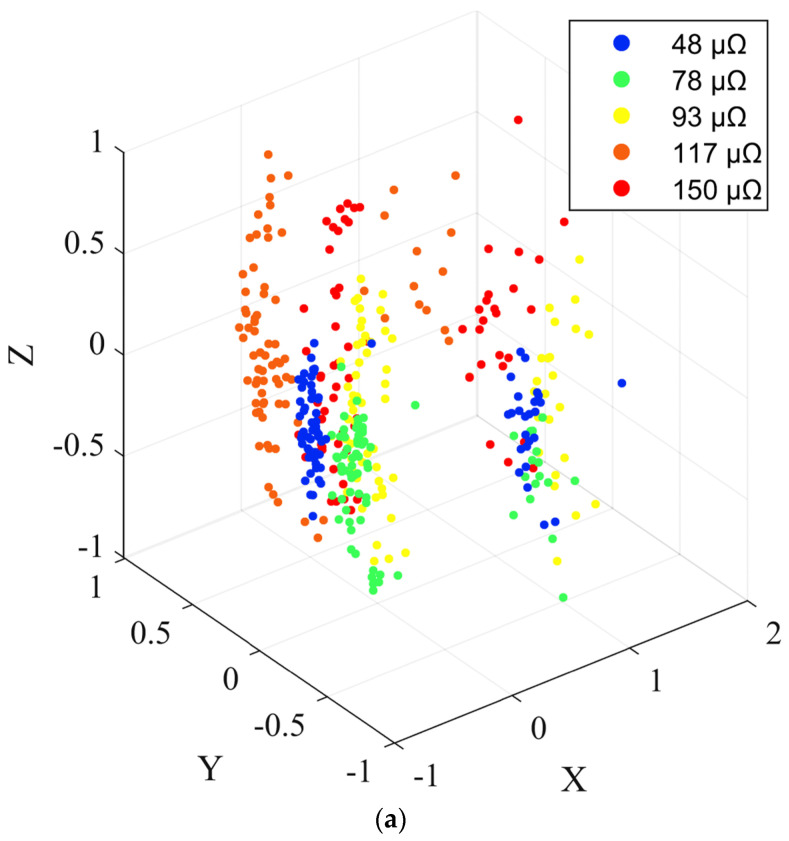

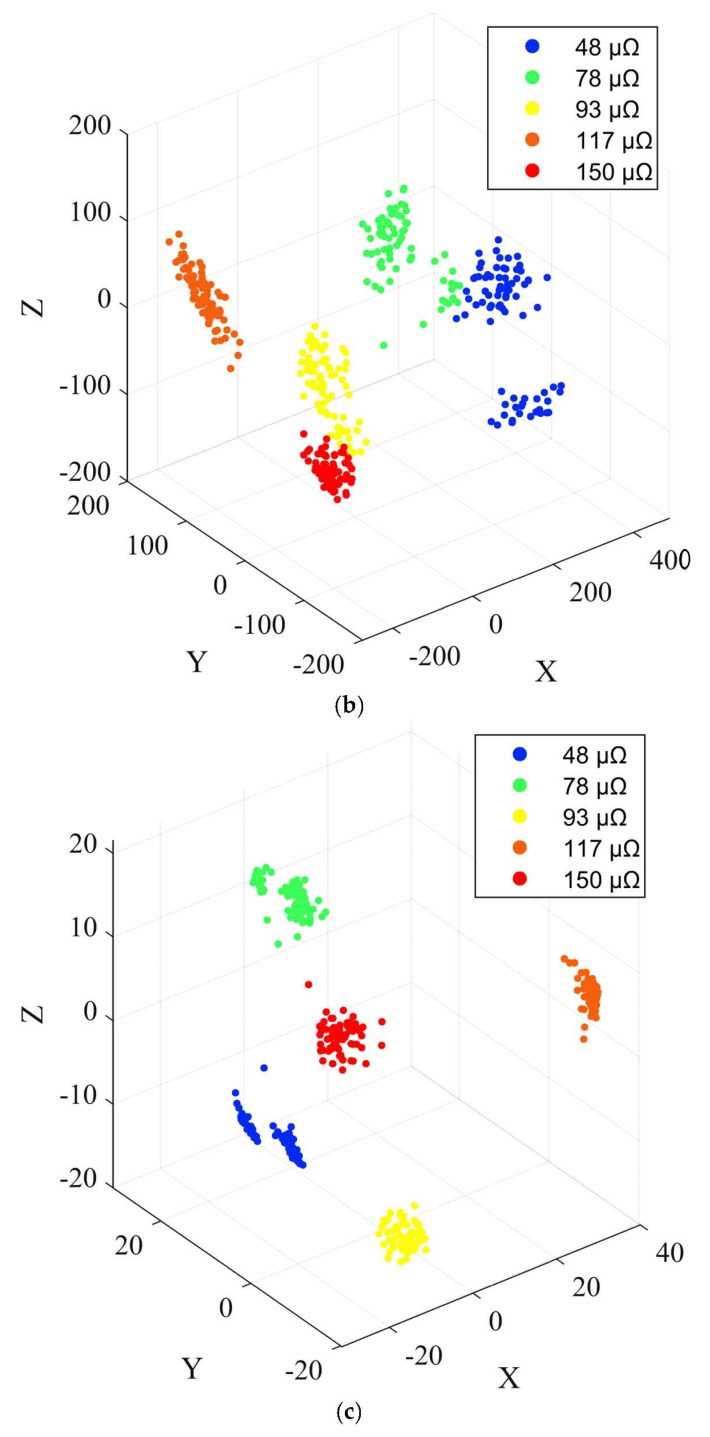
3D display of recognition results: (**a**) input layer, (**b**) hidden layer and (**c**) output layer.

In order to more efficiently and precisely display the results of the HVCB contact state recognition model, 384 sample points in the test set of the HVCB contact state recognition model at a current of 2000 A (At this time, only a hidden layer could be classified.) were selected, and the dimensionality reduction process was carried out by principal component analysis (PCA). Afterward, the classifications of the sample points of the input layer, the hidden layer and the output layer were mapped to a three-dimensional plane. The results are shown in Figure 11. In Figure 11, the x, y and z axes represent the three dimensions after data dimensionality reduction, and there is no specific physical meaning.

It can be observed form Figure 11 that at the input layer, the locations of the sample points were disorganized and scattered throughout the space. After going through a hidden layer, the positions of the sample points began to show a certain pattern, and the data points in the five cases were separated. At the output layer, the data points under different contact resistances were further separated, presenting an ordered distribution in space. This shows that the HVCB contact status recognition model can complete the classification of sample points under different contact resistances, which verifies the effectiveness of the model.

## 5. Conclusions

This paper studied the different states of SF_6_ HVCB contacts while considering various currents for power system applications. The main conclusions of this study are summarized as follows.

With the change in the contact state of the HVCB, the spectrogram of the vibration signal collected by the sensors in the field showed a certain law. The vibration signal frequency of the acquisition point was concentrated at integer multiples of 50 Hz, the range existed in between 50 Hz and 1500 Hz, and the amplitude was the largest at 100 Hz. The vibration was mainly caused by electromagnetic force, and the frequency of the electromagnetic force was 100 Hz. The amplitude of the vibration signal increased with the increase in the interruption current. When the HVCB was in poor contact, the 300-Hz signal amplitude increased.

Considering the amplitude of the signal at a frequency of 50 Hz as the eigenvalue, the signal was used as the eigenvalue to form an eigenvalue matrix, which could fully depict the mechanical vibration characteristics of the SF_6_ HVCB when different contacts were in contact. The contact state recognition model of the HVCB was established by replacing the stochastic gradient descent in the DNN with an Adam algorithm supplemented by BO, which is hyperparameter optimization. At an HVCB current of 1000–3500 A, the model could accurately identify five different contact states. In the experiment, the accuracy rate of the measurements could reach more than 96%. The contact state recognition model proposed in this paper can play a certain role in the condition detection of HVCB and promote the development of intelligent detection technology for future monitoring and diagnosis of faults in HVCBs.

## Figures and Tables

**Table 1:** Parameters of the acquisition system.

Parameters	Value
Measuring range	0.5–3000 Hz
Sensitivity	10.2 mV/(m/s^2^)
Temperature range	From −18 to +66 °C
Weight	4.0 g
Sampling rate	51.2 kHz
Sampling time length	45 s

**Table 2:** Accuracy of status recognition at different currents when fixed parameters are used. (a) When the learning rate is 0.01, the number of hidden layers is 4, and the dropout rate is 0.15. (b) When the learning rate is 0.02, the number of hidden layers is 5, and the dropout rate is 0.25.

Current (A)	Accuracy (%)
(a)	
1000	20.18
1500	22.00
2000	92.65
2500	93.75
3000	97.66
3500	99.21
(b)	
1000	95.05
1500	96.88
2000	96.35
2500	82.68
3000	79.56
3500	84.38

**Table 3:** The search space set in Bayesian optimization.

Hyperparameters	Search Space
Learning rate	[1 × 10^−5^, 5 × 10^−3^]
The number of hidden layer nodes	[128, 256, 512, 1024]
The number of hidden layers	[1, 10]
Dropout rate	[0.1, 0.5]

**Table 4:** Recognition accuracy at different currents.

Current (A)	Accuracy (%)
1000	96.88
1500	99.74
2000	99.87
2500	99.21
3000	100
3500	100

## Data Availability

Not applicable.
